# Supplementary material for: Whey Protein Derived Mouthdrying Found to Relate Directly to Retention Post Consumption but Not to Induced Differences in Salivary Flow Rate
Source: Foods. 2021 Mar 11;10(3):587. doi: 10.3390/foods10030587 (PMC8000321; doi:10.3390/foods10030587)
Supplement: Supplementary file 1 [file foods-10-00587-s001.pdf]

Supplementary Materials: The following are available online at www.mdpi.com/xxx/s1, Figure S1: Viscosity of whey beverages. Figure S2: Summary of volunteers (*n* = 40) gLMS (generalised Labelled Magnitude Scale) familiarisation questions. Table S1: Additional factors (such as sex and saliva flow) influencing volunteers (*n* = 40) liking, effort to consume, attribute perception and appropriateness of attribute level (Just-About-Right, JAR) mean ratings of whey beverages. Figure S3. Additional factors influencing volunteers (*n* = 40) modulating saliva flow perceived mouthdrying. Table S2: Summary of baseline protein concentration (mg/mL) in saliva samples. Figure S4a. Additional factors influencing protein concentration in saliva samples post beverage consumption. Figure S4b. Additional factors influencing volunteers (*n* = 40) perceived mouthdrying.

The viscosity of the samples was measured using a rheometer (Modular Compact Rheometer (MCR) 102, Anton Paar, Graz, Austria) with RheoCompass™ software (Version 1.21, Anton Paar, Graz, Austria). The method was adapted from previous work [1–3] and all analysis was carried out with shear rates increasing logarithmically from 0.001 to 1000 s^{-1} providing 43 data points using a parallel plate (PP50; 50 mm) with a gap size of 1.0 mm and the temperature set at 22 °C. All samples were carefully loaded and allowed to rest for 5-min before any measurements were taken and all analysis was performed using six replicates for each beverage from different batches and whey beverages were considered broadly similar, as outlined in Figure S1.

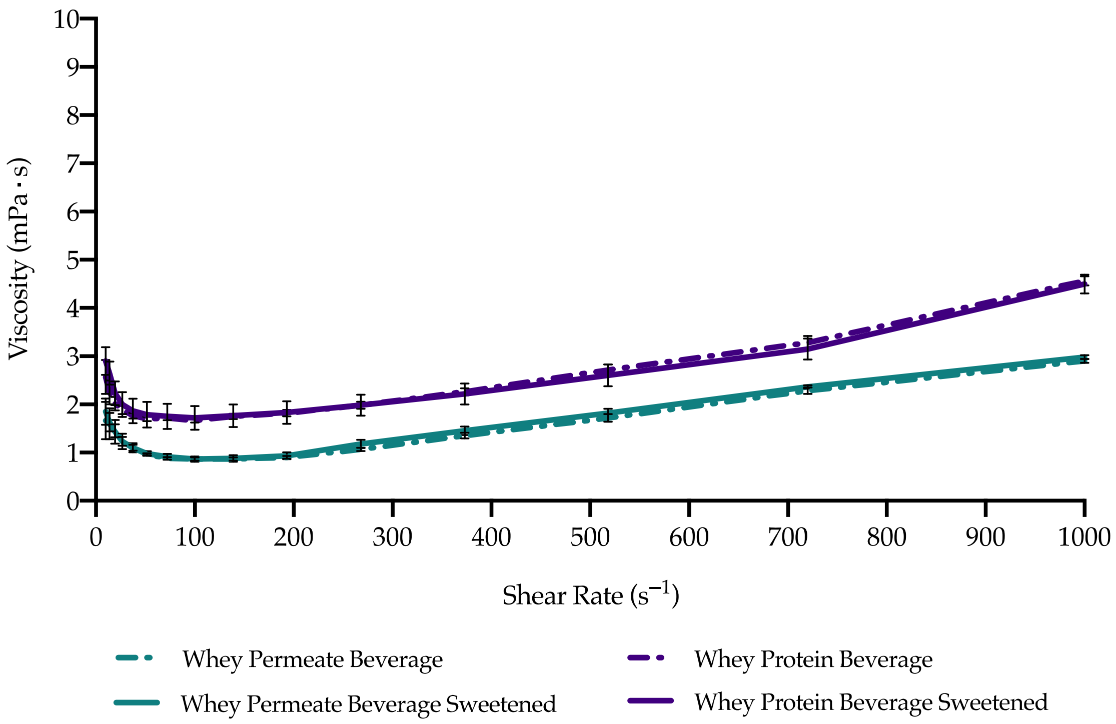

Figure S1. Viscosity (means of six replicates ± standard error) of whey beverages.

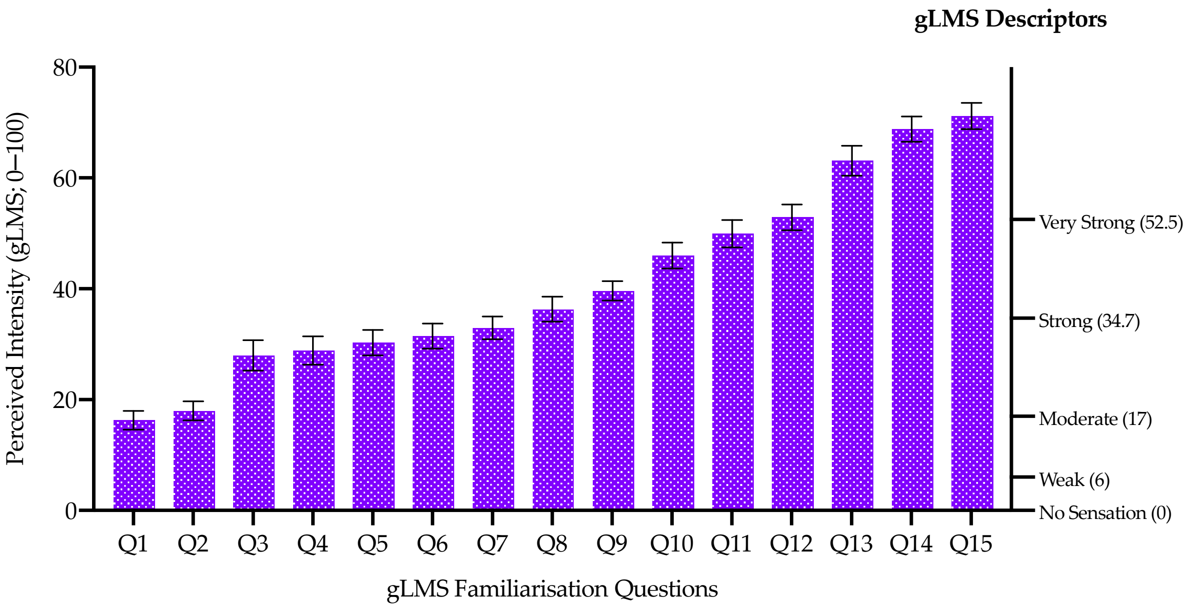

Figure S2. Summary of volunteers (*n* = 40; means of two replicates (visit one and visit two) ± standard error) gLMS familiarisation questions (adapted from [43]). Q1. The brightness of a dimly lit room; Q2. The loudness of a whisper; Q3. The firmness of a handshake; Q4. The warmth of a summer breeze on your face; Q5. The loudness of a conversation; Q6. The cooling of a peppermint candy; Q7. The brightness of a well-lit room; Q8. The sweetness of candy floss; Q9. The bitterness of black coffee; Q10. The sourness of a lemon; Q11. The burn of a chili pepper; Q12. The pain of biting your tongue; Q13. The heat from putting hand in scalding water; Q14. The brightest light you have ever seen and Q15. The loudest sound you have ever heard.

Table S1. Additional factors (such as sex and saliva flow) influencing volunteers (*n* = 40) liking, effort to consume, attribute perception and appropriateness of attribute level (Just-About-Right, JAR) mean ratings (±standard error) of whey beverages.

	**Sex**		**Saliva Flow**	
	Male (*n* = 12)	Female (*n* = 28)	Low Saliva Flow (*n* = 19)	High Saliva Flow (*n* = 20)
Overall Liking	5.9 ± 0.4	5.1 ± 0.3	5.5 ± 0.3	5.4 ± 0.3
Easiness to Drink	3.8 ± 0.2	3.8 ± 0.2	3.9 ± 0.2	3.7 ± 0.2
Easiness to Swallow	3.7 ± 0.2	4.1 ± 0.1	4.0 ± 0.2	3.9 ± 0.2
Sweetness	21.9 ± 3.3	18.6 ± 2.2	19.9 ± 2.9	20.5 ± 2.5
Thickness	15.2 ± 3.1	14.4 ± 2.0	13.5 ± 2.7	16.1 ± 2.3
Mouthdrying	18.9 ± 3.8	22.2 ± 2.5	20.5 ± 3.3	20.6 ± 2.8
JAR Flavour	2.7 ± 0.2	2.9 ± 0.1	2.9 ± 0.1	2.8 ± 0.1
JAR Thickness	2.6 ± 0.2	2.7 ± 0.1	2.6 ± 0.2	2.7 ± 0.1

Individual saliva flow groupings are derived from unstimulated saliva flow only, through quantile 'median' analysis.

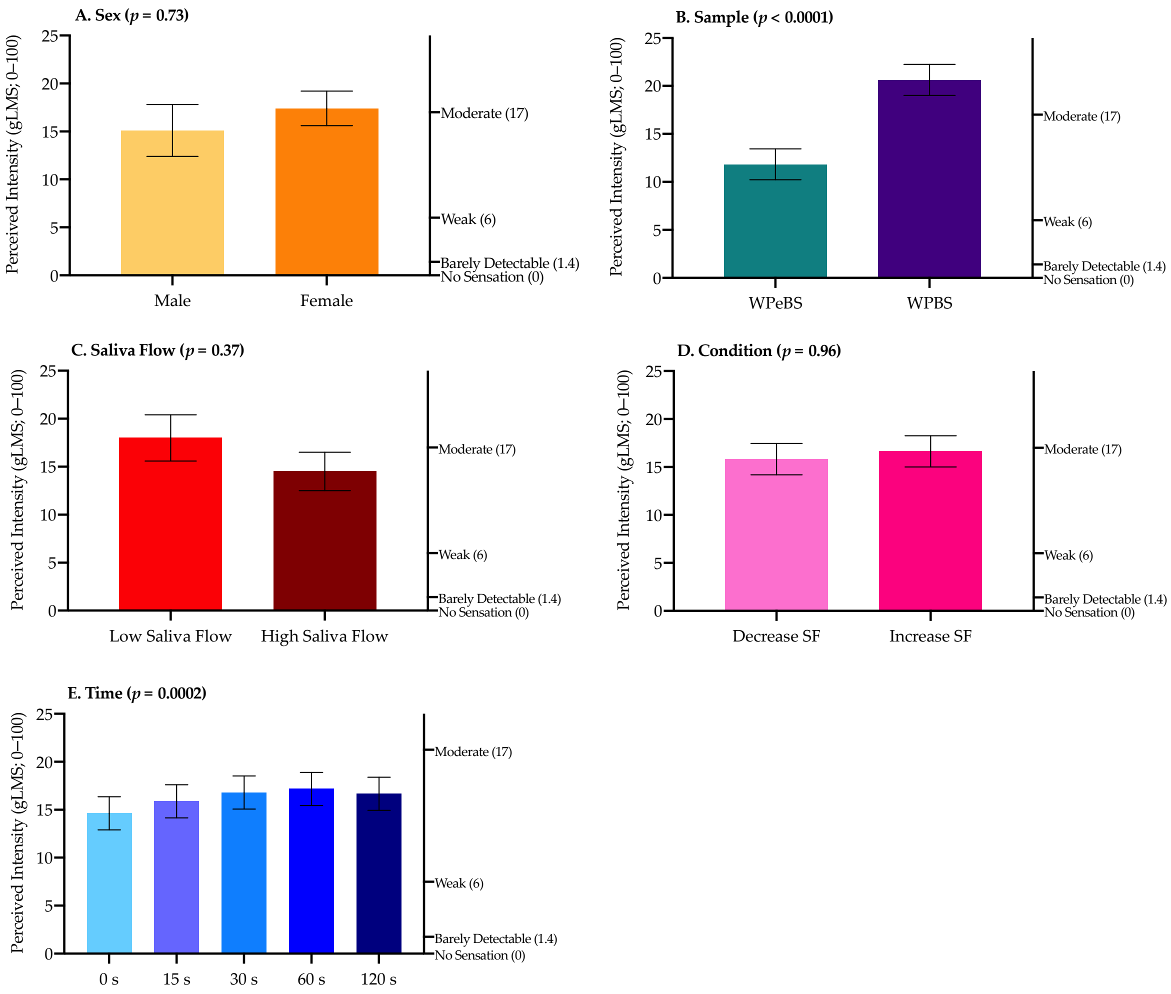

Figure S3. Additional factors (sex, sample, saliva flow, condition & time) influencing volunteers ($n = 40$) modulating saliva flow perceived mouthdrying (± standard error) with relevant p value above each category (WPeBS: whey permeate beverage sweetened; WPBS: whey protein beverage sweetened; SF: saliva flow).

Table S2. Summary of baseline protein concentration (mg/mL) in saliva samples.

	**Overall**	**Sex**	
		Male ($n = 12$)	Female ($n = 38$)
USF	0.94 ± 0.1	0.87 ± 0.2	0.97 ± 0.1
SSF	0.95 ± 0.07	0.84 ± 0.1	1.0 ± 0.1

No significant differences ($p < 0.05$) were reported between groups. USF: unstimulated saliva flow and SSF: stimulated saliva flow.

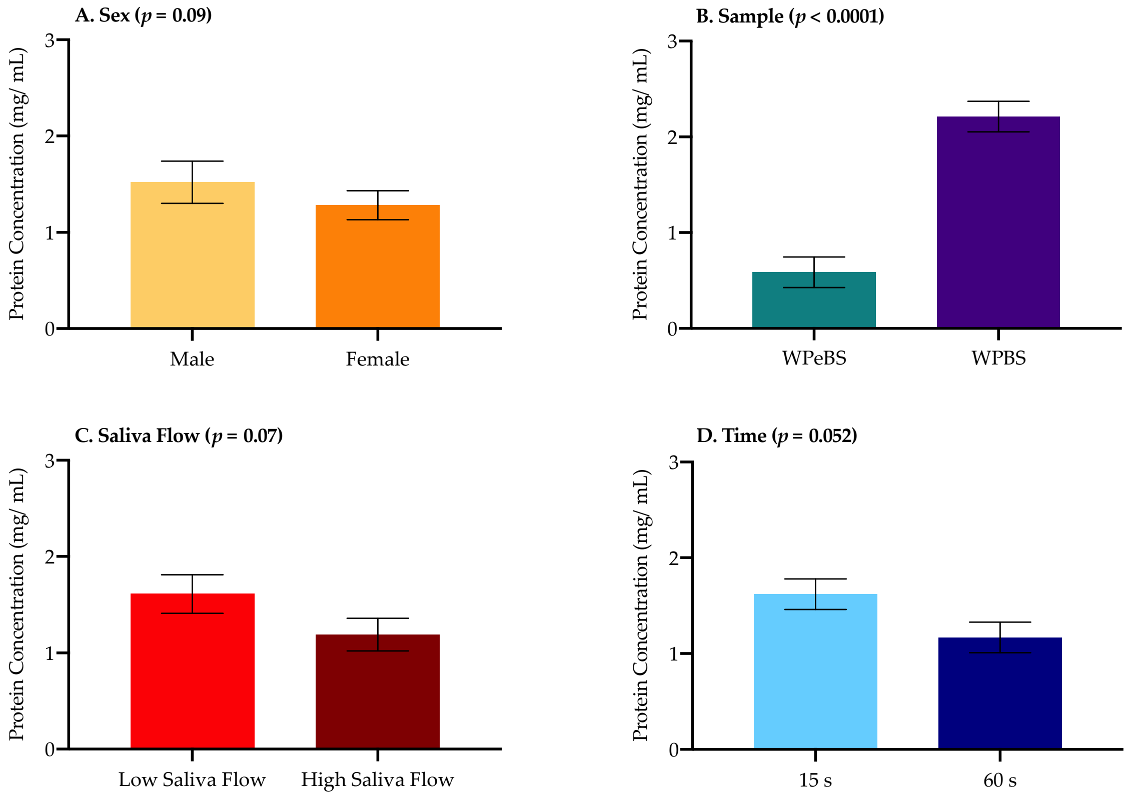

Figure S4a. Additional factors influencing protein concentration (± standard error) in saliva samples post beverage (WPeBS: whey permeate beverage sweetened; WPBS: whey protein beverage sweetened) consumption with relevant p value above each category and a higher value would suggester greater adhesion.

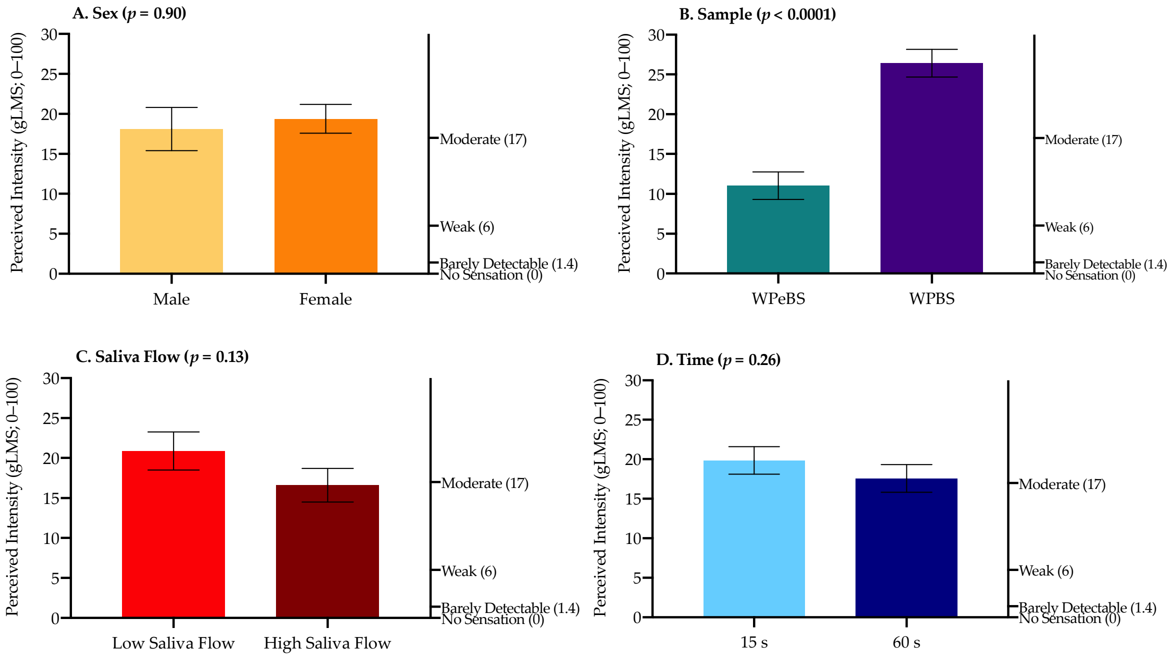

Figure S4b. Additional factors influencing volunteers (n = 40) perceived mouthdrying (± standard error) with relevant p value above each category (WPeBS: whey permeate beverage sweetened; WPBS: whey protein beverage sweetened).

References

1. De Wijk, R.A.; Prinz, J.F.; Janssen, A.M. Explaining perceived oral texture of starch based custard desserts from standard and novel instrumental tests. *Food Hydrocoll.* **2006**, *20*, 24–34.
2. Prinz, J.F.; Huntjens, L.; de Wijk, R.A. Instrumental and sensory quantification of oral coatings retained after swallowing semi-solid foods. *Arch. Oral Biol.* **2006**, *51*, 1071–1079.
3. Moret-Tatay, A.; Rodriguez-Garcia, Marti-Bonmati, E.; Hernando, I.; Hernandez, M.J. Commercial thickeners used by patients with dysphagia: Rheological and structural behaviour in different food matrices. *Food Hydrocoll*. **2015**, *51*, 318–326.
